# MiR-144-5p, an exosomal miRNA from bone marrow-derived macrophage in type 2 diabetes, impairs bone fracture healing via targeting Smad1

**DOI:** 10.1186/s12951-021-00964-8

**Published:** 2021-07-30

**Authors:** Dong Zhang, Yifan Wu, Zonghuan Li, Hairen Chen, Siyuan Huang, Chao Jian, Aixi Yu

**Affiliations:** Department of Orthopedics Trauma and Microsurgery, Zhongnan Hospital of Wuhan University, Wuhan, 430072 Hubei China

**Keywords:** Exosome, Macrophage, MiR-144-5p, Osteoblast, Fracture, Smad1

## Abstract

**Background:**

Patients with diabetes have an increased risk of nonunion and delayed union of fractures. Macrophages have been shown as a key player in diabetic complications. However, it remains obscure how diabetic milieu affects macrophage-derived exosomes and its implications on osteogenic differentiation of BMSCs. In this study, we aim to define the impact of diabetic milieu on macrophage-derived exosomes, role of extracellular vesicles in intercellular communication with BMSCs, and subsequent effects on osteogenic differentiation and fracture repair.

**Results:**

The osteogenic potential and the ability of fracture repair of exosomes derived from diabetic bone marrow-derived macrophages (dBMDM-exos) were revealed to be lower, as compared with non-diabetic bone marrow-derived macrophages (nBMDM-exos) in vitro and in vivo. Interestingly, miR-144-5p levels were sharply elevated in dBMDM-exos and it could be transferred into BMSCs to regulate bone regeneration by targeting Smad1. In addition, the adverse effects of dBMDM-exos on the osteogenic potential and the ability of fracture repair were reversed through the suppression of miR-144-5p inhibition in vitro and vivo.

**Conclusions:**

The results demonstrated an important role of exosomal miR-144-5p in bone regeneration, offering insight into developing new strategy for the improvement of fracture healing in patients with diabetes mellitus.

**Graphic Abstract:**

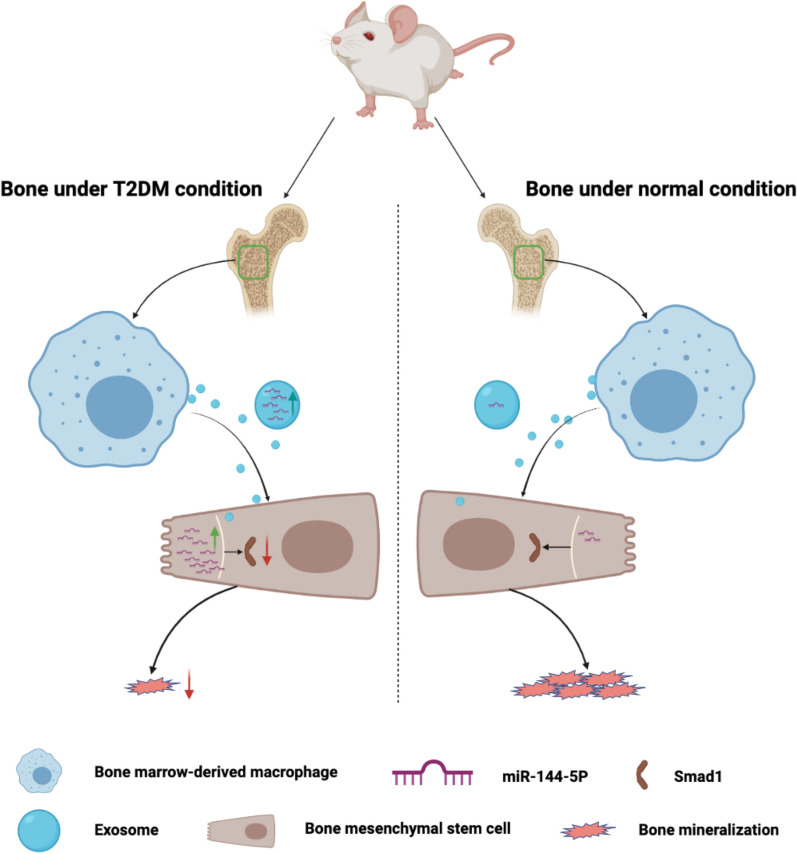

**Supplementary Information:**

The online version contains supplementary material available at 10.1186/s12951-021-00964-8.

## Background

Diabetes mellitus (DM) is a major health problem imposing serious personal and societal healthcare burdens worldwide [1]. Uncontrolled diabetes leads to multiorgan pathology, which ultimately causes disability and reduces life expectancy [2]. Over the past decades, a series of consistent studies in animals and humans have consistently demonstrated that DM adversely affects fracture-healing by disturbing the balance of bone homeostasis, and there is a higher risk of delayed union and nonunion with a doubling of the time to healing of the fracture in diabetes when compared with non-diabetes [3–5].

It is increasingly recognized that impaired bone repair and regeneration in DM is regarded as the result of abnormal crosstalk between macrophage and bone mesenchymal stem cells (BMSCs) that disrupts osteogenic differentiation and fracture healing [6–10]. Macrophages diffusely distributed in the body secrete various bioactive substances, receptors and enzymes that mediate cell to cell communication, and it has been regarded as a crucial aspect of diabetic complications [8–12]. Previous studies have demonstrated that bone marrow-derived macrophages (BMDMs) are situated closely adjacent to BMSCs, and shape the bone microenvironment via secreting various functional biomolecules [11–13]. In addition, BMDMs have been shown to be in adverse pathological status caused via DM, and secrete osteogenesis-impeded factors including IL-1, TNF-α, iNOS and so on, thus impairing osteogenic differentiation and increasing the probabilities of an uncontrolled regulation of fracture repair [14–19]. These factors, however, neither entirely represent the macrophages-BMSCs crosstalk under diabetic disorder nor completely account for the mechanisms underlying the impaired bone repair and regeneration under diabetic condition in DM.

Exosomes are 50–100 nm diameter extracellular vesicles secreted via various cell types, and derive from endosomes; they can carry abundant bioactive substances, such as noncoding RNA, mRNA, DNA, protein and other molecules [20]. Recent studies have demonstrated that they effectively mediate intercellular and interorgan communications via delivering cargos containing specific miRNAs [21]. Some miRNAs have been revealed to participate in the regulation of proliferation, migration, differentiation and apoptosis of BMSCs through modulating the expression of target genes at the post-transcriptional level, indicating that miRNAs are important regulators of bone repair and regeneration [22–24]. And there is evidence that exosomal miRNAs are capable of being stably transferred from the bone microenvironment and into BMSCs, and contribute to the process of osteogenic differentiation and fracture healing [25–28]. As such, exosomal miRNAs represent key regulators of fracture repair with significant roles at various stages of the repair process. Indeed some studies have showed that exosomal miRNAs derived from macrophages are able to influence the fracture healing via crosstalk with BMSC differentiation in vitro and in vivo [29]. Rather, the influence of BMDMs-derived exosomal miRNAs, role of DM, and their crosstalk with BMSCs remain undefined. Therefore, we aimed at evaluating the in vitro and in vivo effects of diabetic milieu on BMDMs-derived exosomal miRNAs, alterations in intercellular crosstalk with BMSCs, and influence on osteogenic differentiation and fracture repair. And it may provide effective approaches and potential therapeutic targets for the treatment of impaired fracture healing in DM.

## Results

### Evaluation of the T2DM rat model

**Fig. 1:**
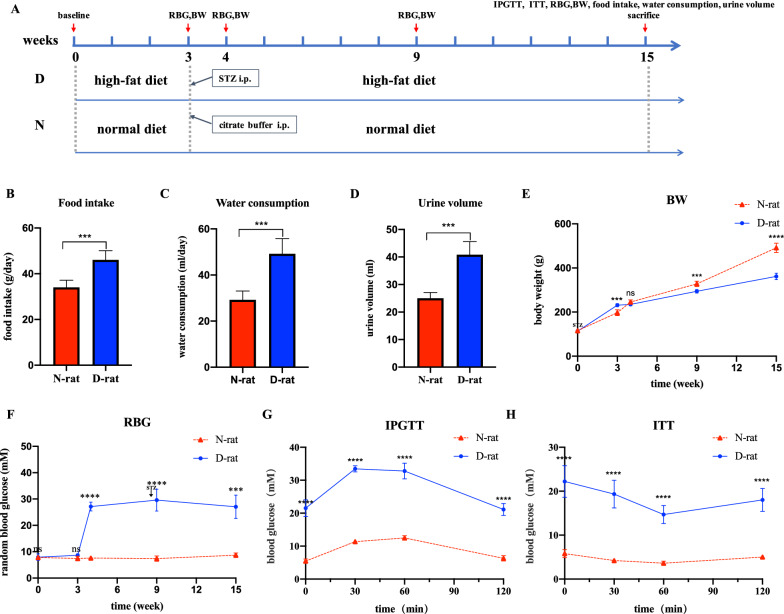
Establishment and evaluation of HFD/STZ-induced T2DM rat models. **a** Scheme of animal treatments. **b–d** Food intake, water consumption and urine volume of normal and T2DM rats at 12 weeks after STZ injection. **e–f** Body weight and random blood glucose levels at the time points before HFD, before STZ injection, one week after STZ injection, 6 weeks after STZ injection and 12 weeks after STZ injection. **g–h** Blood glucose levels during IPGTT and ITT in normal versus T2DM groups at 12 weeks after STZ injection. Data are presented as the mean ± SD. n = 5, ***p < 0.001, ****p < 0.0001

Our data showed that all HFD/STZ-induced rats showed 2 type diabetes mellitus. As shown in Fig. 1, there were significantly higher food intake, water consumption and urine output in T2DM group compared with the control group(*P* < 0.05). Meanwhile, rats in the T2DM group were thinner than control group (*P* < 0.05). All these are the typical symptoms of type 2 diabetes mellitus. As presented in Fig. 1, at three days after STZ injection, the random blood glucose level in T2DM group was significantly higher compared to normal group and remained higher than 16.7 mmol/l until the end of the experiment. In addition, to investigate glucose tolerance and insulin sensitivity in two groups, the IPGTT and ITT were performed. The data showed that HFD/STZ-induced rats displayed hyperglycemia in comparison with control rats during 120 min after glucose administration (Fig. 1). The ITT study revealed that blood glucose levels were rapidly decreased in normal group after insulin injection, whereas the blood glucose levels were reduced slowly or even not reduced in T2DM group within 30 min. These results indicated that the successful establishment of type 2 diabetes mellitus rat models with insulin resistance and hyperglycemia.

### nBMDM-exos and dBMDM-exos can be internalized by BMSCs

**Fig. 2:**
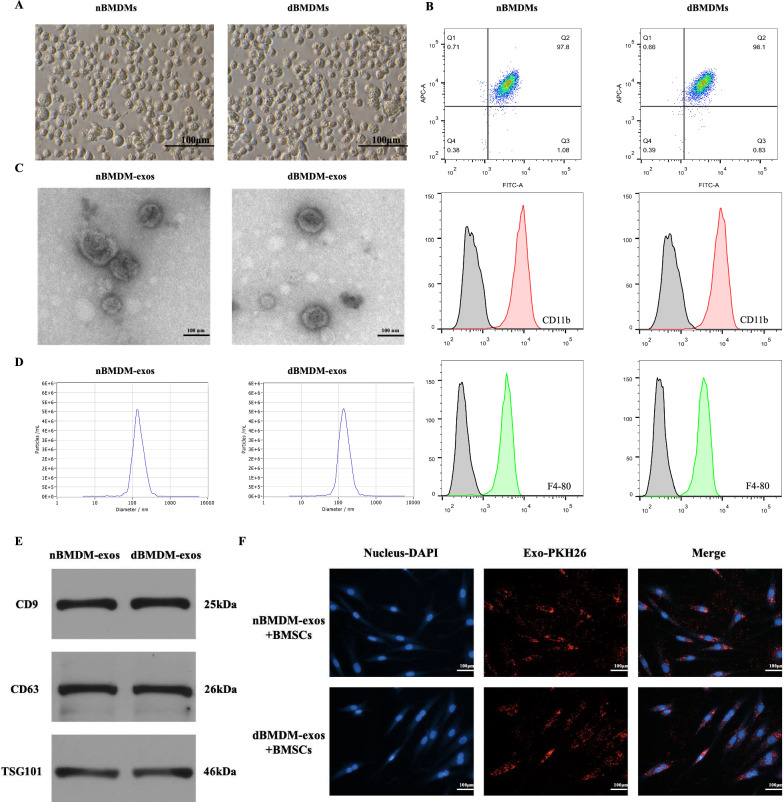
Identification of BMDMs and their exosomes. **a** Morphology of the BMDMs under microscopy. **b** BMDMs surface markers F4-80 and CD11b were analyzed by flow cytometry. **c** Morphology of exosomes determined by TEM. **d** Particle size distribution of exosomes measured by DLS. **e** Exosome surface markers CD9, CD63 and TSG101 were examined by Western blotting. **f** BMDMs-derived exosomes were internalized by BMSCs. All experiments were repeated three times and shown are representative data

BMDMs were thus obtained from femurs and tibias of rats in T2DM group or normal group. The BMDMs were characterized by morphology and flow cytometry studies. Cells showed round-like shape and were positive for F4-80 and CD11b, confirming the successful isolation of BMDMs from rats (Fig. 2a, b and Additional file 1: Fig. S1). The extracted exosomes derive from nBMDMs and dBMDMs were examined by using transmission electron microscopy (TEM), dynamic light scattering (DLS) and Western blotting. TEM images showed that the particles displayed a cup- or round- shaped morphology (Fig. 2c). The particle sizes, as revealed by DLS analysis, ranged from 30 to 200 nm (Fig. 2d). In addition, the specific surface markers of exosomes including CD9, CD63 and TSG101 were validated by using the Western blotting analyses (Fig. 2e). These data support the successful isolation of exosomes from nBMDMs and dBMDMs. Furthermore, an exosomes uptake assay was performed, and the results demonstrated that nBMDM-exos and dBMDM-exos could be internalized by BMSCs (Fig. 2f).

### dBMDM-exos hinder BMSCs osteogenesis differentiation

**Fig. 3:**
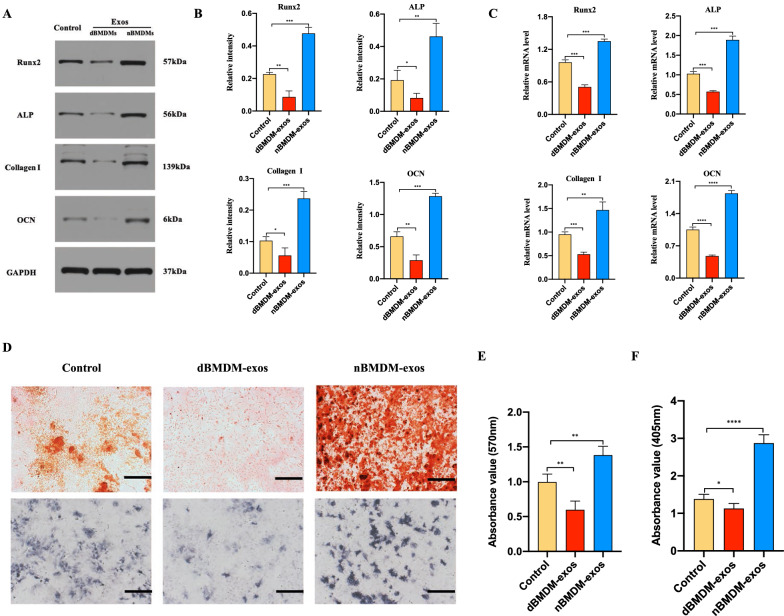
dBMDM-exos hinder BMSCs osteogenesis differentiation in vitro. **a–c** The protein and mRNA levels of some osteogenic genes in BMSCs treated with PBS, nBMDM-exos or dBMDM-exos were determined by Western blotting and RT-qPCR analyses, respectively. **d** The proportion of mineralization in treated BMSCs was shown by Alizarin red staining and ALP staining assays. **e, f **The Alizarin red and ALP staining were quantitatively determined. Data are presented as the mean ± SD (n = 3 per group), *p < 0.05, **p < 0.01, ***p < 0.001, ****p < 0.001

We then determined the effects of nBMDM-exos and dBMDM-exos on the BMSCs osteogenesis differentiation in vitro. After BMSCs were cocultured with 200 µg/ml nBMDM-exos, 200 µg/ml dBMDM-exos or equal quantities of PBS respectively, their osteogenesis-related genes including RUNX2, ALP, collagen I, OCN were measured via Western blotting and qRT-PCR methods. As shown in Fig. 3a–c, the protein and mRNA levels of RUNX2, ALP, collagen I, OCN were downregulated in dBMDM-exos groups in comparisons with other two groups. Furthermore, as shown by ALP staining and Alizarin red staining, the proportion of mineralization was increased by nBMDM-exos, but decreased by dBMDM-exos when compared with PBS group (Fig. 3d–f). These data indicated that dBMDM-exos may inhibit the BMSCs osteogenic differentiation.

## dBMDM-exos impair femoral fractur healing in a rat model

**Fig. 4:**
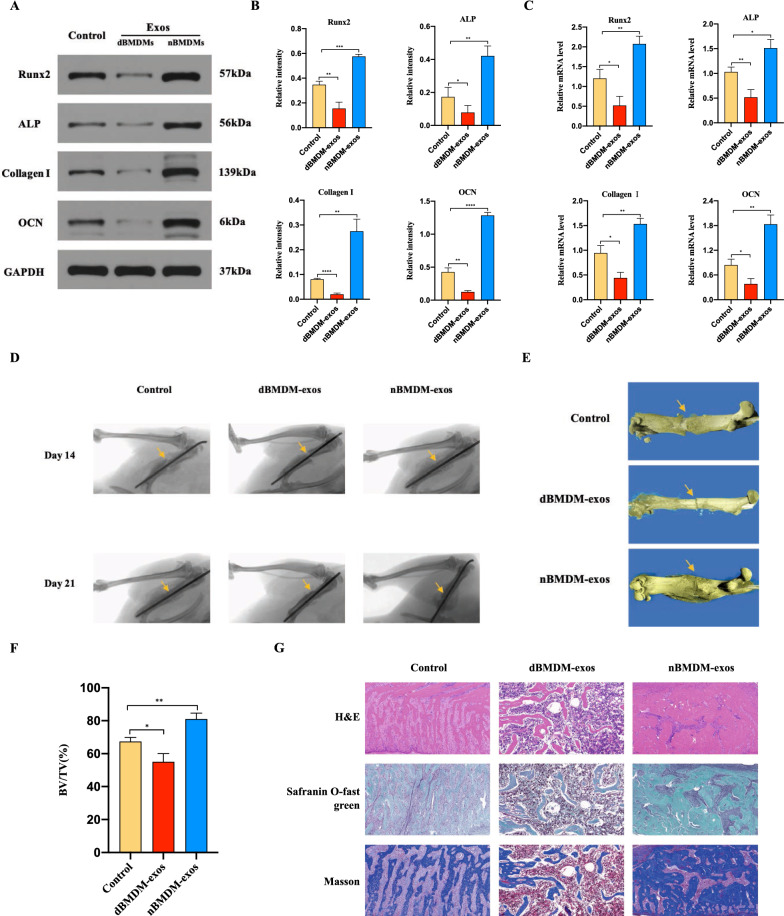
dBMDM-exos impair femoral fractur healing in vivo. **a–c** The osteogenic genes at the mRNA and protein levels in fractures region locally injected with PBS, nBMDM-exos or dBMDM-exos for 21 days were measured via qRT-PCR and western blotting. **d** Representative X-ray images of femurs on 14 and 21 days after surgery from different treated groups. **e** Representative 3D micro-CT images of femurs among different treatment groups on day 21 after surgery. **f** The BV/TV data on 21 days after surgery were quantitatively analyzed based on micro-CT results. **g** H&E, safranin O-fast green staining and Masson of the femurs on day 21 after surgery. Data are presented as the mean ± SD. n = 5 rats/group, *p < 0.05, **p < 0.01, ***p < 0.00,****p < 0.001

To investigate the role of dBMDM-exos in the bone repair and regeneration in vivo, we used a rat transverse femur shaft fracture model treated with PBS, nBMDM-exos or dBMDM-exos. Western blotting and qRT-PCR analyses of samples from fracture region showed that the protein and mRNA levels of RUNX2, ALP, collagen I, OCN were significantly lower in dBMDM-exos group compared with PBS or nBMDM-exos group (Fig. 4a-c). In addition, the fracture repair at the fracture site was evaluated through X-ray and micro-CT examinations. The results indicated that compared with the PBS or nBMDM-exos group, the dBMDM-exos group a thinner callus volume and larger fracture gap (Fig. 4d, e). Quantitative analysis of micro-CT data demonstrated that the bone volume/total volume (BV/TV) values of the dBMDM-exos group were significantly decreased when compared with other groups (Fig. 4f). As shown in Fig. 4g, histology images for H&E, safranin O-fast green, Masson showed that there was a visible hindrance in the fracture healing of the femur in the rats treated with dBMDM-exos compared with rats in other two groups. Our results demonstrated that dBMDM-exos impaired femoral fractur healing in the rat models.

### The identification of differentially expressed miRNAs in nBMDM-exos and dBMDM-exos

**Fig. 5:**
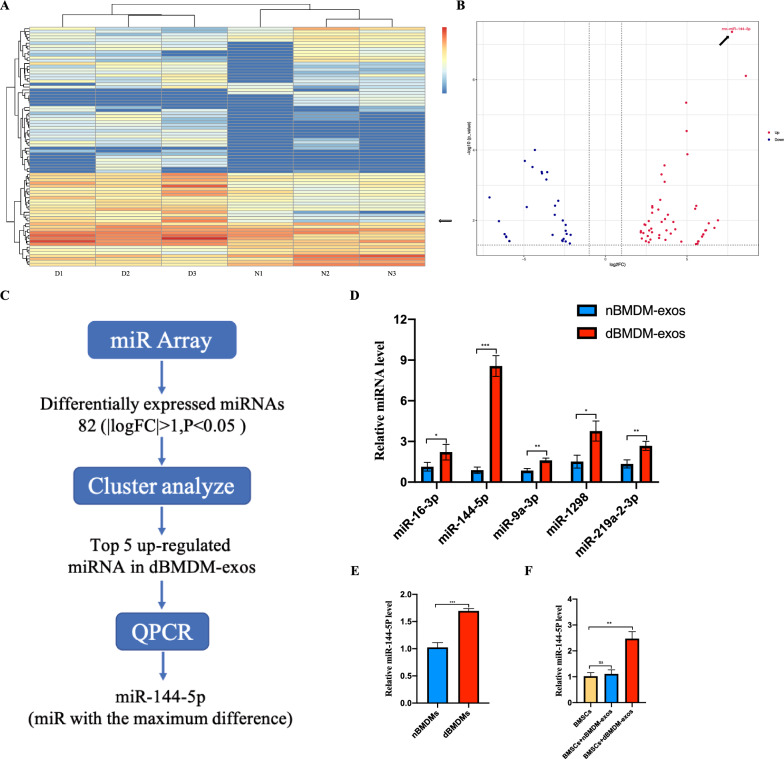
MiR-144-5p was sharply increased in dBMDM-exos and it can be transferred to BMSCs via exosomes. **a** The heat map showed that differently expressed miRNAs(|logFC|>1, P < 0.05. **b** Volcano plot of miRNAs expressed differently in dBMDM-exos or nBMDM-exos. **c** The schematic diagram of microRNA screening and selection. **d **qRT-PCR analyses confirmed that, among the top 5 most upregulated miRNAs in the dBMDM-exos, miR-144-5p was increased to the greatest degree. **e** Comparison of miR-144-5p levels between nBMDMs and dBMDMs, as determined with qRT-PCR assay. **f** The miR-144-5p expression levels among groups following BMSCs treated with PBS, nBMDM-exos or dBMDM-exos. Data are presented as the mean ± SD. *p < 0.05, **p < 0.01, ***p < 0.001

Becuase exosomal miRNAs play important roles in bone repair and regeneration, we further identified the differentially expressed miRNAs between nBMDM-exos and dBMDM-exos by conducting miRNA sequencing study. And as shown in Fig. 5a, b, the Volcano plot and heat map analysis displayed that a number of miRNAs were significantly upregulated in dBMDM-exos. The top five miRNAs including miR-16-3p, miR-144-5p, miR-9a-3p, miR-1298, miR-219a-2-3p altered to the greatest degrees were validated via qRT-PCR, among which miR-144-5p was the most upregulated in dBMDM-exos (Fig. 5d). Based on the RT-qPCR results, we also found that miR-144-5p was significantly enriched in dBMDMs when compared with nBMDMs, which was consistent with that in exosomes (Fig. 5e). Furthermore, these exosomes were co-cultured with BMSCs, the RT-qPCR results showed that BMSCs treated with dBMDM-exos exhibited significantly increased expression of miR-144-5p (Fig. 5f). Together, these results suggest that miR-144-5p is elevated in dBMDM-exos and dBMDM-derived exosomal miR-144-5p can be internalized by BMSCs.

## miR-144-5p suppresses BMSCs osteogenesis differentiation

**Fig. 6:**
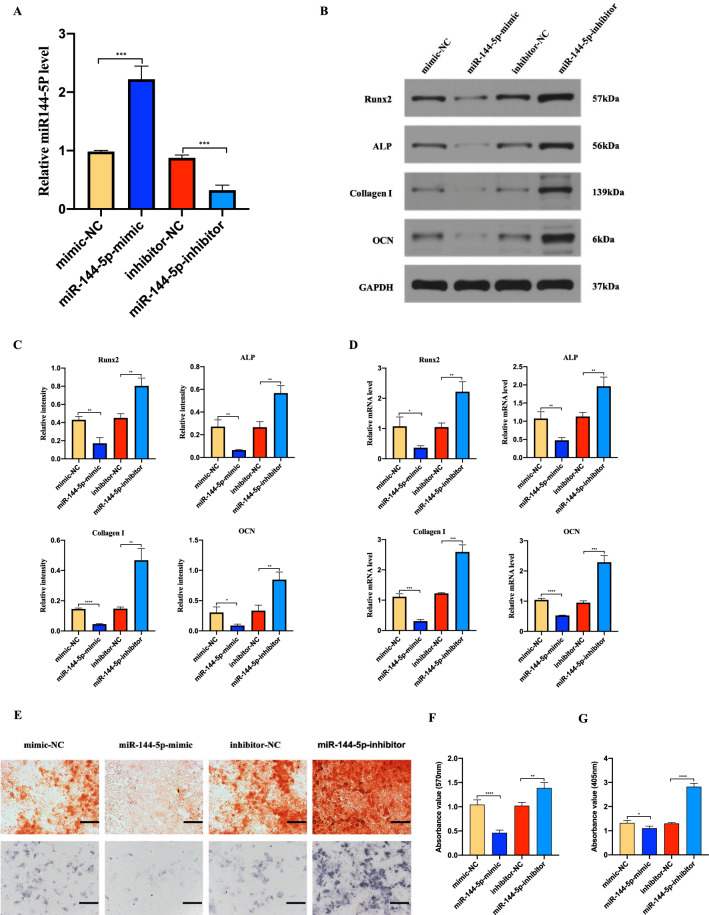
Role of miR-144-5p in BMSCs osteogenesis differentiation. **a** Levels of miR-144-5p in BMSCs transfected with mimic-NC, miR-144-5p-mimic, inhibitor-NC or miR-144-5p-inhibitor were analyzed by qRT-PCR. **b–d** The osteogenic genes at the mRNA and protein levels in the transfected BMSCs were confirmed via qRT-PCR and Western blotting. **e** The proportion of mineralization in the transfected BMSCs was shown by Alizarin red staining and ALP staining assays. **f**, **g** The statistical data of Alizarin red staining and ALP staining. Data are presented as the mean ± SD, and all experiments were repeated three times. *p < 0.05, **p < 0.01, ***p < 0.001, ****p < 0.001

To investigate the effect of miR-144-5p on BMSCs osteogenesis differentiation, BMSCs were transfected with miR-144-5p-mimic, mimic-NC, miR-144-5p-inhibitor or inhibitor-NC respectively. qRT-PCR results showed that miR-144-5p-mimic increased the levels of miR-144-5p in BMSCs, while that with miR-144-5p-inhibitor significantly decreased the levels of miR-144-5p in BMSCs (Fig. 6a). Next, the mRNA and protein levels of RUNX2, ALP, collagen I, OCN in the indicated BMSCs were determined by using qRT-PCR and Western blotting. And a significantly decreased expression in these osteogenesis related genes was observed in miR-144-5p-mimic group, but an increased expression happened in miR-144-5p-inhibitor group (Fig. 6b–d). Moreover, as shown by Alizarin red and ALP staining, the mineralization was increased after miR-144-5p inhibition, while decreased following miR-144-5p overexpression (Fig. 6e–g).

## miR-144-5p regulates osteogenesis differentiation via targeting Smad1

**Fig. 7:**
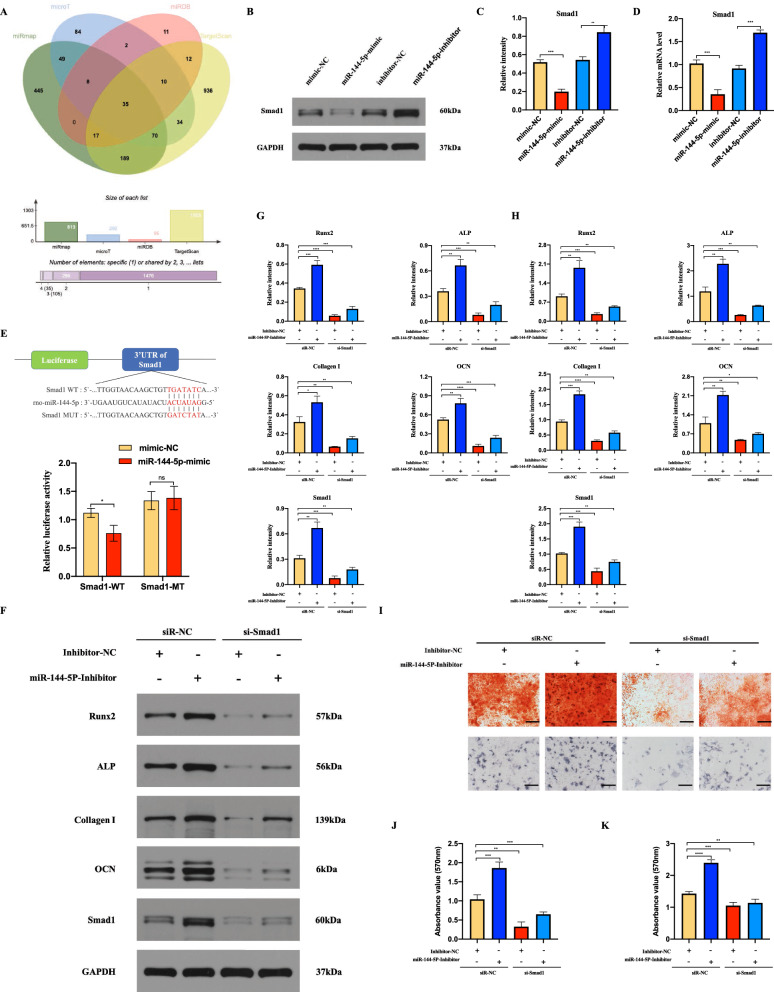
MiR-144-5p impairs BMSCs osteogenesis differentiation via targeting Smad1. **a** Venn diagram of the targeting genes of miR-144-5p predicted by microT, TargetScan, miRDB, and miRmap. **b–d** Western blotting and qRT-PCR analysis of Smad1 protein and mRNA levels, respectively, in BMSCs transfected with mimic-NC, miR-144-5p-mimic, inhibitor-NC or miR-144-5p-inhibitor **e **WT and mutated miR-144-5p recognition sites within the Smad1 3’ UTR were constructed for the dual-luciferase reporter assay and the results demonstrated Smad1 as a direct target gene for miR-144-5p. **f–h** After BMSCs were con-transfected with miR-144-5p-inhibitor or si-Smad1; the mRNAs and proteins of Smad1, RUNX2, ALP, collagen Ι and OCN were determined using qRT-PCR and Western blotting. **I** the mineralization in BMSCs following different treatments was measured via Alizarin red staining and ALP staining. **j–k** The statistical data of Alizarin red staining and ALP staining. Data are presented as the mean±SD, and all experiments were repeated three times. *p < 0.05, **p < 0.01, ***p < 0.001, ****p < 0.001

To further evaluate the mechanism by which miR-144-5p regulates the BMSCs osteogenic differentiation, four bioinformatics tools (microT, TargetScan, miRDB, and miRmap) were used to identify putative miR-144-5p target (Fig. 7a). Among these candidate target genes, Smad1 playing a key role in bone morphogenetic protein (BMP) signaling pathway was finally selected as target gene for further investigations. As shown in Fig. 7b-d, we found that overexpression of miR-144-5p significantly downregulated the expression of Smad1 at both the mRNA and protein levels, while the opposite effects were observed after the inhibition of miR-144-5p. Furthermore, we used a luciferase reporter assay to evaluate the interactions between miR-144-5p and the 3’-UTR of Smad1 in BMSCs. As shown in Fig. 7e, the luciferase activity of wt-Smad1 was decreased by miR-144-5p overexpression. However, the alterations of the luciferase activity were abolished in mut-Smad1. After confirming that miR-144-5p could regulate Smad1, the miR-144-5p/ Smad1 axis on BMSCs osteogenic differentiation was further estimated. BMSCs were co-transfected with miR-144-5p-inhibitor and si-Smad1, and then the osteogenic related genes and the mineralization were determined. And the results demonstrated that the mRNA and protein levels of RUNX2, ALP, collagen Ι, OCN and Smad1 were significantly increased via miR-144-5p inhibition, but the opposing effects on the above mRNAs and proteins were exerted via si-Smad1(Fig. 7f–h). And the effect of miR-144-5p-inhibitor could be partly reversed by si-Smad1 (Fig. 7f–h). In the meanwhile, the mineralization levels were measured via Alizarin red and ALP staining, and the results showed that it was also promoted via miR-144-5p-inhibitor, while inhibited by si-Smad1; the effect of miR-144-5p-inhibitor could also be partially reversed via si-Smad1(Fig. 7i–k). These results suggest that miR-144-5p regulates osteogenesis differentiation via targeting Smad1.

### **dBMDM-derived exosomal miR-144-5p suppresses the bone repair and regeneration in vitro and vivo**

**Fig. 8:**
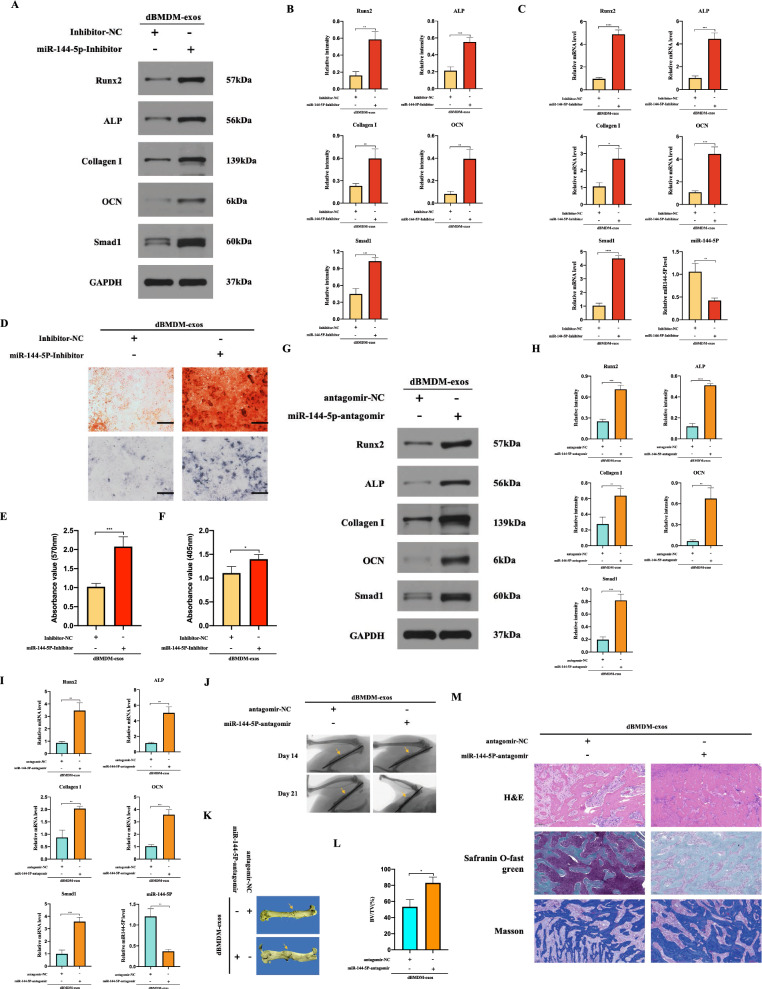
The effects of dBMDM-exos on bone repair and regeneration in vivo and vitro can be reversed through the suppression of miR-144-5p. **a**, **b** The protein levels of Smad1 and some osteogenic genes in groups following BMSCs treated with dBMDM-exos + inhibitor-NC or dBMDM-exos + miR-144-5p-inhibitor in vitro. **c** The levels of the osteogenic genes and miR-144-5p in treated BMSCs were measured via qRT-PCR. **d.** Alizarin red staining and ALP staining results in BMSCs with different treatments. **e–f** The statistical data of Alizarin red staining and ALP staining. **g–i** Levels of RUNX2, ALP, collagen I, OCN, Smad1 and miR-144-5p was determined using qRT- PCR or western blotting after dBMDM-exos + antagomir-NC or dBMDM-exos + miR-144-5p-antagomir was locally injected around the fracture site. **j–k** The bone formation in femur fracture was observed via X-ray images on 14, 21 days after surgery and micro-CT approach on 21 days after surgery. **l** The BV/TV data on 21 days after surgery were quantitatively analyzed based on micro-CT results. **m** H&E, safranin O-fast green staining and Masson of the femurs on 21 days after surgery. Data are presented as the mean±SD, all cell experiments were repeated three times, and n = 5 rats/group. *p < 0.05, **p < 0.01, ***p < 0.001, ****p < 0.001

After transfecting the miR-144-5p-inhibitor or inhibitor-NC into BMSCs which were co-cultured with dBMDM-exos, the expression of miR-144-5p were significantly decreased in miR-144-5p-inhibitor group, but significantly increased osteogenic related genes, Smad1 and the mineralization were detected when compared with inhibitor-NC group (Fig. 8a–f). To further define the impact of dBMDM-derived exosomal miR-144-5p on fracture healing, a transverse femur shaft fracture model injected with dBMDM-exos were treated with antagomir-NC or miR-144-5p-antagomir. On day 21 post-surgery, samples from fracture region were collected for qRT-PCR and Western blot analyses, and the results confirmed that miR-144-5p was downregulated, while that of Smad1 was upregulated in miR-144-5p-antagomir group in comparison with antagomir-NC group (Fig. 8g–i). In addition, the protein and mRNA expression of osteogenesis related genes were also increased in miR-144-5p-antagomir group compared with antagomir-NC group (Fig. 8g–i). The X-ray images and micro-CT studies revealed a statistically decreased fracture gap and a larger callus volume happened in miR-144-5p-antagomir group, while the opposite effect happened in antagomir-NC (Fig. 8j, k). Further analysis of BV/TV offered a similar result (Fig. 8L). Moreover, histological analysis showed that there was an obvious enhancement in the fracture healing in miR-144-5p-antagomir group compared with antagomir-NC group (figure m). These results indicated that dBMDM-derived exosomal miR-144-5p could suppress BMSCs osteogenesis differentiation and impair the femoral fracture healing process, and alternation of miR-144-5p could rescue the adverse effects of dBMDM-exos on bone repair and regeneration in vitro and vivo.

## Discussion

DM affects hundreds of million people worldwide and impaired bone healing is an important DM-related complication which remains a threat to human health and life. Given the quickly growing incidence of diabetes especially for T2DM, it is important to improve bone healing exposed to diabetic milieu. While the mechanism of impaired fracturing healing under diabetic condition has been widely studied, no investigations focused on the macrophages-BMSCs crosstalk relied on exosomes under diabetic disorder. Recently, mounting studies have shown that exosomes can transfer miRNAs between cells, resulting in functional changes in the receiving cells and affecting bone regeneration [26, 27]. In this research, we find that the DM changes the profile of exosomal miRNAs in BMDMs, and increased exosomal miR-144-5p in dBMDMs can impair bone regeneration via targeting smad1 in vivo and vitro, which hold great potential to be a novel therapeutic target.

It’s acknowledged that BMDMs play an essential role in modulating the osteogenesis ability of BMSCs under DM condition, while most previous studies ascribe the results to enzymes or growth factors secreted via BMDMs. In recent years, exosomes have attracted much attention based on the ability that proteins, metabolites, and nucleic acids could be transported via exosomes into recipient cells effectively affect their biological process, and it holds potentials to identify unknown cellular and molecular mechanisms in tissue regeneration and various disease from the perspective of cell-to-cell communication locally and between organs [21]. Furthermore, evidence have shown that macrophage-derived exosomes and their cargo might play the important role in the pathological processes of DM as well as its related complication. Wei et al. indicated that adipose tissue macrophage-derived exosomes transfer miRNAs to insulin target cells, and modulate in vivo and in vitro insulin sensitivity which can cause insulin resistance [30]. Consistent with the above findings, another study demonstrated that adipose tissue macrophage-derived exosomal miR-29a could regulate obesity-associated insulin resistance [31]. And an elegant experiment on diabetic mice showed that the pathogenesis of diabetes nephropathy was characterized by the abnormal cell–cell communication kidney-infiltrating macrophages and glomerular mesangial cells, and this cell interaction was mediated via macrophages-derived exosomes [32]. In addition, Govindappa et al. found that exosomes from BMDMs under DM condition significantly increase inflammatory and profibrogenic responses in fibroblast (in vitro) and cardiac fibrosis in mice, which reveals a novel regulatory mechanism of diabetic cardiac fibrosis and dysfunction [33]. On the basis of these findings, therefore, we assumed that exosomes derived from BMDMs exposed to DM could communicate with BMSCs via miRNAs cargo to control bone repair and regeneration in rats with DM.

To test our hypothesis, we first successfully obtained and identified BMDMs from rats with or without T2DM. Then, exosomes were isolated from the supernatant of either nBMDMs or dBMDMs, and was identified via TEM, DLS and western blotting. In accordance with the reports that exosomes can be internalized the neighboring or distant cells to modulate the function of recipient cells, our findings suggest that both of exosomes from two groups can be taken up into BMSCs, and the osteogenesis ability of BMSCs was significantly impaired after incubation with dBMDM-exos when compared with other two groups. Furthermore, our in vivo studies displayed that the process of fracture repair and regeneration in a transverse femur shaft fracture model was hindered with local injection treatment of dBMDM-exos. Given the fact that exosomes participate in intercellular communication via containing numerous cargo molecules, and microRNAs are deeply involved in the regulation of the osteogenesis of BMSCs, we focus on the effect of exosomal miRNAs derived from BMDMs deposed to DM condition on the fracture healing in vitro and in vivo. Therefore, we further to identify the underlying miRNA that contribute to the negative regulation of bone repair and regeneration in dBMDM-exos group, and miR-144-5p was identified as the most upregulated miRNA in dBMDM-exos using miRNA sequencing and qRT-PCR. Furthermore, our findings also revealed that miR-144-5p was significantly upregulated in BMSCs treated with dBMDM-exos, which laid an important foundation for further researches. More importantly, our in vivo studies demonstrated that the protein levels of osteogenesis markers including RUNX2, ALP, collagen I and OCN were significantly reduced after the overexpression of miR-144-5p in BMSCs.

Four bioinformatics algorithms including microT, TargetScan, miRDB, and miRmap were used to get the potential target genes of miR-144-5p. Among all these candidate target genes, Smad1 attracts our attention. It’s acknowledged that bone morphogenetic protein (BMP) is a member of the transforming growth factor-beta (TGF-β) superfamily which plays an essential role in the process of bone repair and regeneration; and as an immediate downstream molecules of BMP receptors, Smad1 is involved in the regulation of BMSCs osteogenesis via affecting BMP signal transduction [34–36]. And several studies have confirmed that Smad1 can be as the target gene of non-coding RNA. Based on these evidences, the relationship between miR-144-5p and Smad1 was verified using luciferase assays, and further research revealed that miR-144-5p negatively regulates the osteogenic differentiation via decreasing the expression of Smad1 in BMSCs. In addition, findings have also demonstrated that miR-144-5p-inhibitor is able to promote the osteogenic differentiation, while this effect could be partly attenuated by si-Smad1. More importantly, we further observed that the inhibition of miR-144-5p could affect the negative effect of dBMDM-exos on bone repair and regeneration in vitro and vivo. All these results indicate that dBMDM-derived exosomal miR-144-5p can hinder the fracture healing via decreasing the expression of Smad1.

## Conclusions

Taken together, our results suggest that dBMDM-derived exosomal miR-144-5p can be transferred into BMSCs and reduce the bone repair and regeneration by suppressing the expression of Smad1 in vivo and vitro. These findings shall provide new insights into the molecular mechanism of impaired bone repair in T2DM patients and development of new remedy strategies.

## Methods

### T2DM induction

Ten male Sprague-Dawley rats at age 5 weeks and weighting 100–130 g were randomly divide into two groups: the T2DM group (n = 5) and the normal group (n = 5). The protocols of establishing T2DM rat model have been described in detail previously [37–42]. Just after 3 days of adjusting to the new environment, the rats in T2DM group were fed with a high-fat diet (HFD) containing 60 % fat for 3 weeks, then they received STZ via intraperitoneal (IP) injection (40 mg/kg in citrate buffer with pH 4.5, Sigma, USA). The normal group fed with the normal pelleted diet for 3 weeks were intraperitoneally injected with an equal volume of citrate buffer (pH 4.5). Blood sample collected from the tail was consecutively measured for blood glucose levels. One week later, rats with more than three random blood glucose level > 16.7 mmol/l were classified as being T2DM rats. Then, to generate a long-term T2DM complication rat model, all rats in two group were returned to their cages and provided with free access to their original diets (the high-fat or control diet) and normal drinking water for 12 weeks. To evaluate this model, the food intake, water consumption, and volume of excreted urine in two groups were measured at the time point before HFD and 12 weeks after STZ injection; the body weight and random blood glucose level was recorded at the time point before HFD, before STZ injection, one week after STZ injection, 6 weeks after STZ injection and 12 weeks after STZ injection. At 12 weeks after STZ injection, IPGTT was performed. All animals were fasted overnight and given 1.5 g/kg glucose via IP injection. Tail blood samples were taken at 0, 30, 60, and 120 min for measurement of blood glucose level. In addition, ITT was carried out by administering insulin (0.75 IU/kg, ip), then blood glucose was measured at 0, 30, 60, and 120 min after insulin injection. During all the period of the experiment, rats with blood glucose levels below 10 mmol/l were considered non-diabetic and between 10 mmol/l and 16.7 mmol/l were excluded from the study. At 12 weeks after STZ injection, animals were euthanized for the isolation of BMDMs.

### Cell culture

BMDMs rats were isolated from rat models and cultured following the methods previously [43]. Briefly, bone marrow cells were flushed via PBS from the femurs and tibias in non-diabetic and diabetic groups after 12 weeks of citrate buffer or STZ injection, respectively. To obtain the BMDMs, cells were cultured in RPMI-1640 medium (without glucose) supplemented with 10 % fetal bovine serum (FBS) 1 % penicillin/streptomycin, and differentiated with 25 ng/ml rat GM-CSF for 7 days, with fresh culture medium supplemented on the 4th day. And the glucose concentration of medium was adjusted using a glucose solution; to mimic the non-diabetic and diabetic conditions in vitro, cells from non-diabetic group were cultured in the medium supplemented with glucose (5 mM), and the glucose concentration of medium used in diabetic group was 30 mM. After 7 days of culture, BMDMs were harvested for identification via flow cytometric analysis and cultured for the isolation of exosomes. BMSCs were isolated and cultured as described previously, and identified by the morphology and flow cytometric analysis [44]. The BMSCs in the passage 3–5 were used in the following experiments. The maintaining medium consists of α-MEM supplemented with 10 % FBS and 100 U/mL penicillin/streptomycin (Invitrogen). All cells were maintained in a humidified environment with 5 % CO_2_ at 37 °C.

### Flow cytometry

BMDMs were analysed by flow cytometry. BMDMs from two groups were detected with antibodies against F4-80 (Abcam) and CD11b (Abcam). Results were analyzed by using Flowjo software.

### Exosome isolation and identification

Briefly, after reaching 70–80 % confluence, BMDMs were washed three times via PBS and cultured in the media with exosome-depleted FBS for 48 h. And the cell culture supernatant was collected and centrifuged at 100,000*g* for 6 h at 4 °C. After centrifugation, samples were washed using PBS and continue undergoing ultracentrifugation at 100,000*g* for 20 min 4 °C. Then the pelleted exosomes were collected in 15 ml of PBS and centrifuged at 4000*g* in ultra-clear tubes to concentrate the volume to approximately 200 µl for following experiments. The morphology of exosomes was observed via transmission electron microscopy (TEM; HITACHI, HT7700). And nanoparticle tracking analysis (NTA; ZetaView, Particle Metrix, Meerbusch, Germany) was used to analysis the diameter distributions of both dBMDM-exos and nBMDM-exos. In addition, specific exosome surface markers like CD9, CD63, TSG101 were identified via Western blot.

### Exosome uptake assay

Briefly, BMSCs uptake of the BMDM-derived exosomes was observed through labeling the exosomes using the PKH26 Fluorescent Cell Linker Kit (Sigma) according to the manufacturer’s instructions. Firstly, 20 µg exosomes was mixed with 1 ml PKH26 dye solution, incubated for 20 min at 25 °C, and was washed three times with PBS. Then, they were collected and resuspended via centrifuged at 110,000*g* for 20 min at 4 °C. Finally, PKH26-labeled exosomes were added to DAPI-labeled BMSCs and cultured for 12 h before being observed via a confocal fluorescence microscope.

### MiRNA sequencing

Samples were processed according to methods described previously [45]. Total RNA was isolated from exosomes using TRIzol reagent (Invitrogen, CA, USA) according to the manufacturer’s protocol. The quality and quality of RNA samples were assessed with a NanoDrop ND-1000 and Agilent 2100 Bioanalyzer, respectively. Total RNA from each sample was used to prepare miRNA library using NEBNext® Multiplex Small RNA Library Prep Set for Illumina® (NEB) according to the Illumina small RNA sample preparation protocol. In short, adapters were ligated to the 3′ and 5′ ends of all miRNAs, and cDNA was synthesized by reverse transcription, then the cDNA samples were amplified by PCR Reaction. After library amplification, a cleanup of the miRNA library was performed to choose the fragment ranged from 135 to 155 bp (corresponding to ∼15–35 nt small RNAs). Next, the quality and quantity of the library was carefully verified in automated electrophoresis, performed on Agilent 2100 Bioanalyzer. And the libraries were denatured as single-stranded DNA molecules, captured on Illumina flow cells, amplified in situ as clusters and finally sequenced for 50 cycles on an Illumina NextSeq 500 (Illumina, CA, USA) system according to the manufacturer’s instructions. Differentially expressed miRNA were then identified at a criteria of fold change > 1.0 or < 1.0 and a p value < 0.05, and demonstrated via the heat map and the volcano plot.

### BMSCs osteogenic differentiation

The BMSCs were seeded into 6-well plates and was used for osteogenic differentiation induction via a specific osteogenic induction medium (OIM, OriCell, Cyagen Biosciences, Guangzhou, China). The entire inducing process lasted 14 days. For evaluating the effect of exosomes on osteogenic differentiation, 200 μl exosomes (200 µg/mL) and equal volume PBS were added to osteogenic induction medium and refreshed every three days. In addition, BMSCs were transfected with siSMAD1, miR-144-5p mimic, miR-144-5p inhibitor and NC via Lipofectamine 3000 (Invitrogen) when cells confluency reached 70–80 %. The reagents of siRNA, miR-144-5p-mimic, miR-144-5p-inhibitor and miR-144-5p-NC were synthesized by RiboBio (Guangzhou, China), and the sequences are listed in Table 1. To evaluate the level of osteogenic differentiation, alkaline phosphatase (ALP) staining, alizarin red staining, quantitative reverse transcription polymerase chain reaction (qRT-PCR) and western blot were performed on day 14.

**Table. 1:** The sequences of primers used for PCR studies

MicroRNAs or gene name	Primer (5′ to 3′)	Primer sequence
MiR-144-5p	ForwardReverse	GGGGGACTTACAGTATATGATGACTCAACTGGTGTCGTGGAGTC
Runx2	ForwardReverse	ACTCTGCCGAGCTACGAAATGGGGACCGTCCACTGTCACTT
ALP	ForwardReverse	TGGACCTCATCAGCATTTGGGAGGGAAGGGTCAGTCAGGTT
collagen I	ForwardReverse	CCGTGACCTCAAGATGTGCCGAACCTTCGCTTCCATACTCG
OCN	ForwardReverse	CCAGCGACTCTGAGTCTGACAAAACGGTGGTGCCATAGATGC
Smad1U6	ForwardReverseForwardReverse	TGTGTCACCATTCCTCGCTCCCACTCGCTTATAGTGGTAGGGCCTGCTTCGGCAGCACATAACGCTTCACGAATTTGCGT
GAPDH	ForwardReverse	AACAGCAACTCCCATTCTTCCTGGTCCAGGGTTTCTTACTCC

### ALP activity and mineralization assessment

To identify the osteogenic differentiation, BMSCs were induced for 14 days with different treatment, and measured via ALP staining and alizarin red staining. According to the protocol, BMSCs were washed three times with PBS, fixed with 4 % paraformaldehyde for 20 min, and then stained with ALP staining or alizarin red staining for 30 min at 25 °C. After staining, the BMSCs were washed with PBS and observed using microscopy. Absorbance was then measured at 570 nm or 405 nm.

### Luciferase reporter assay

After BMSCs were added to 24-well plates at a density of 0.5−2 × 10^5^ each well for 24 h, dual-luciferase vectors (pGL6-miR-Smad1-WT-3′ UTR, pGL6-miR-Smad1-Mut-3’UTR) were transfected into them together with miR-144-5p mimics or mimic-NC. And the luciferase activity was examined using Dual-Luciferase Reporter Assay System (Promega) after 48 h post-transfection. Ultimately, the results were normalized to Renilla luciferase activity.

### The transverse femur shaft fracture establishment and treatment

Rat transverse femur shaft fracture model was created as follows. All rats were conducted under general anesthesia with intraperitoneal injection of 60 mg/kg ketamine hydrochloride before surgical procedures. After the lower limb of the rat was shaved and disinfected, a 3-cm incision was created under sterile conditions. To expose the femurs, the soft tissues were pushed aside while the overlying periosteum was carefully preserving. And a transverse femur shaft fracture was conducted using an oscillating saw before a Kirschner’s wire with 1-mm in diameter was inserted into the femur for intramedullary fixation. Finally, the subcutaneous tissue and the skin were closed using 5−0 nylon suture. After fracture, 200 µL PBS and equivalent volumes of nBMDM-exos, dBMDM-exos, dBMDM-exos + NC-antagomir, dBMDM-exos + miR-144-5p-antagomir were respectively injected around the fracture site at the time point 1, 3, 5, 7 day after surgery. And X-ray images of fracture femurs were taken at day 14 and 21 after surgery. In addition, at 21 days after operation, samples were harvested for histological analysis, micro computed tomography (micro-CT), qRT-PCR and western blot analysis.

### Radiographic analysis

A small animal in vivo imaging system (Bruker Xtreme BI) was used to obtain X-ray images to observe fracture regions. After rats were subjected to internal fixation removal at 21 days after surgery, all femurs were fixed with 4 % paraformaldehyde for 24 h at 4 °C. Then samples were scanned via performing a micro-CT system (SkyScan1276, Bruker, Belgium) at a resolution of 40 μm with 63 kV and 200 µA. After scanning, we constructed 3D structures of femurs and calculated the new bone volume/total volume (BV/TV) to assess bone regeneration in the fracture site.

### qRT-PCR analysis

Total RNA was extracted from exosomes, BMSCs induced for 14 days or callus tissues harvested from fracture regions at 21 days after surgery using TRIpure Extraction Reagent (EP013, ELK Biotechnology). The cDNA was reverse transcribed using EntiLink™ 1st Strand cDNA Synthesis Kit (Eq. 003, ELK Biotechnology) following the manufacturer’s instructions. And the qRT-PCR for mRNAs and miRNAs was performed on a StepOne™ Real-Time PCR System (Life technologies) using EnTurbo™ SYBR Green PCR SuperMix (EQ001, ELK Biotechnology). The relative expression levels of mRNA or miRNA were normalized to those of GAPDH or U6 and evaluated using the 2^−ΔΔ^CT method. The primers used for qRT-PCR are listed in Table 1.

### Western blot analysis

Total protein was isolated from exosomes, BMSCs induced for 14 days or callus tissues harvested from fracture regions at 21 days after surgery using RIPA (Aspen) in accordance with the manufacturer’s protocol. After the concentrations of protein samples were detected using BCA (Aspen), equal amounts of protein samples were separated by sodium dodecyl sulfate-polyacrylamide gel electrophoresis (the 15 % concentration for OCN; the 12 % concentration for CD9,CD63;the 10 % concentration for TSG101, RUNX2, ALP and Smad1; the 8 % concentration for collagen I), transferred to a polyvinylidene fluoride (PVDF) membrane, and incubated with 5 % bovine serum albumin for 1 h at 25 °C. And PageRuler™ Prestained Protein Ladder (ThermoFishe, 26,617) was used as a molecular weight marker. Next, the membranes were incubated overnight at 4 °C with primary antibodies specific for CD9 (1:1000, Abcam, ab92726), CD63 (1:500, Santa, sc-5275), TSG101 (1:1000, Abcam, ab125011), RUNX2 (1:1000, Abcam, ab236639), ALP (1:1000, Bioss, bsm-52252R), collagen I (1:500, Abcam, ab260043), OCN (1:500, Santa, sc-390,877), Smad1 (Abcam100, Abcam, ab53745) and GAPDH (1:10,000, Abcam, ab37168). Then, they were stained with appropriate secondary antibodies at 1:2,000 for 30 min. The visualization of the reacting bands was performed via ECL reagent (Thermo Fisher Scientific). And the protein bands were quantified using AlphaEaseFC (Alpha Innotech, San Leandro, CA) software. The original WB image of the osteogenic related proteins was shown in Additional file 2: Fig. S2.

### Histological analysis

The femurs of rats from different groups were collected 21 days after surgery. Samples were fixed with buffered paraformaldehyde for 48 h, and then decalcified with 20 % EDTA at 25 °C for 25 days. The samples were embedded in paraffin, sectioned along the longitudinal axis, and stained with H&E, safranin O-fast green, Masson for histological analysis. Sections were then imaged with a microscope.

### Statistical analysis

Values were presented as mean ± SD, and analyzed with GraphPad Prism 8.0 (Graph- Pad Software, CA, USA). All experiments were repeated at least three times. Student’s t-test was used to analyze the two independent groups. A value of P < 0.05 was considered as statistically significant.

## Supplementary Information

**Additional file 1: Fig. S1.** BMDMs were identified via flow cytometry.

**Additional file 2: Fig. S2.** The original WB image of the osteogenicrelated proteins.

## Data Availability

All data generated or analyzed during this study are included in this published article.

## Abbreviations

D: T2DM group
N: Normal group
RBG: Random blood glucose
BW: Body weight
IPGTT: Intraperitoneal glucose tolerance test
ITT: Insulin tolerance test
i.p.: Intraperitoneal
STZ: Streptozocin
BMDM: Bone marrow-derived macrophages
nBMDMs: Bone marrow-derived macrophages from rats under normal condition
dBMDMs: Bone marrow-derived macrophages from rats under diabetic condition
Exos: Exosomes
dBMDM-exos: Exosomes derived from dBMDMs
nBMDM-exos: Exosomes derived from nBMDMs
BMSCs: Bone mesenchymal stem cells
TEM: Transmission electron microscopy
DLS: Dynamic light scattering
Runx2: Recombinant runt related transcription factor 2
ALP: Alkaline phosphatase
OCN: Osteocalcin
