# Supplementary figures and images for: MiR-144-5p, an exosomal miRNA from bone marrow-derived macrophage in type 2 diabetes, impairs bone fracture healing via targeting Smad1
Source: J Nanobiotechnology. 2021 Jul 30;19:226. doi: 10.1186/s12951-021-00964-8 (PMC8327443; doi:10.1186/s12951-021-00964-8)

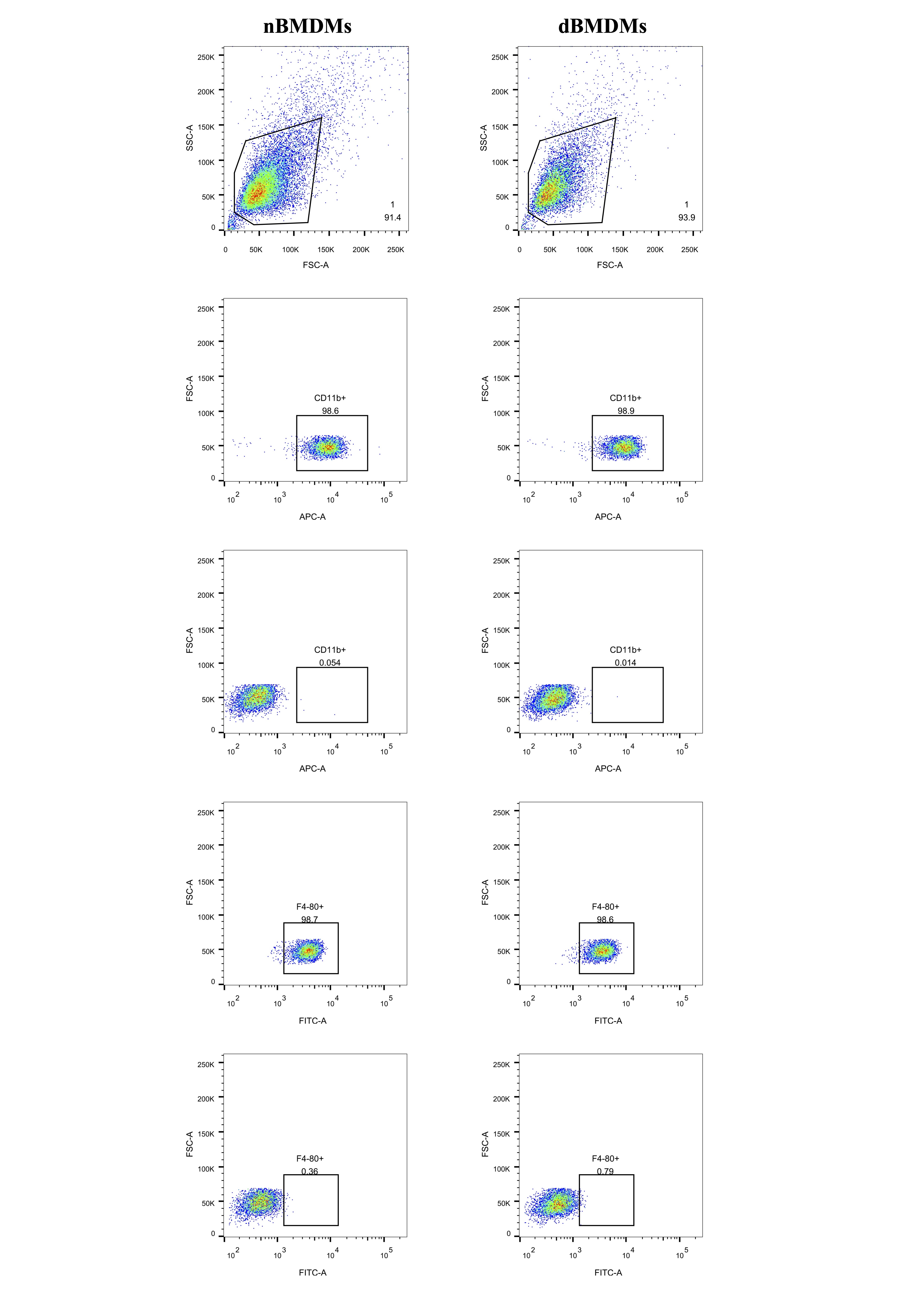

Supplement: Supplementary file 1 — Additional file 1: Fig. S1. BMDMs were identified via flow cytometry. [file 12951_2021_964_MOESM1_ESM.jpg]
